# Draft genome sequence of *Acinetobacter pittii* carrying the ISAba43 mobile genetic element isolated from a urinary tract infection sample in Dhaka, Bangladesh

**DOI:** 10.1128/mra.00863-25

**Published:** 2025-11-12

**Authors:** Md. Shiful Islam Sujan, Akhi Khanom, Dewan Zubaer Islam, Sawda Binte Monir, Md. Mehedi Hasan, Md. Firoz Ahmed, Salma Akter, Nafisa Azmuda, Nihad Adnan

**Affiliations:** 1 Department of Microbiology, Jahangirnagar University 115523, Dhaka, Bangladesh; 2 Department of Microbiology, Stamford University 421920, Dhaka, Bangladesh; University of Maryland School of Medicine, Baltimore, Maryland, USA

**Keywords:** *Acinetobacter pittii*, urinary tract infections, mobile genetic elements

## Abstract

ISAba43 is a mobile genetic element (MGE) previously reported in *Acinetobacter baumannii* carrying antibiotic-resistance genes. The draft genome of a multidrug-resistant *Acinetobacter pittii* strain containing this MGE, isolated from a urinary tract infection (UTI) sample, is reported, highlighting the clinical relevance of the isolate.

## ANNOUNCEMENT

*Acinetobacter pittii* is a Gram-negative, non-motile, aerobic coccobacillus (1, 2) that is increasingly associated with hospital-associated infections (3), with outbreaks reported worldwide and a higher prevalence in Southeast Asia (1, 2).

This study presents the draft genome of *A. pittii* Vector-K19, obtained from a diagnostic laboratory (4), with ethical approval from the Biosafety, Biosecurity, and Ethical Committee of Jahangirnagar University (Ref No: BBEC, JU/M-2022/2 [4]). The diagnostic laboratory isolated the bacteria from a urine sample and initially isolated it on a MacConkey agar (Oxoid, UK) plate. From the initial culture plate, at the Department of Microbiology of Jahangirnagar University, the isolate was subcultured on nutrient agar (Oxoid, UK) at 37°C for 24 h for identification using conventional biochemical tests, the API (Analytical Profile Index) 20E system (BioMérieux, France), the VITEK-2 system (BioMérieux, France), and the MALDI-ToF Biotyper (Bruker Scientific LLC, USA). A pure colony from the nutrient agar was aseptically transferred to 5 mL nutrient broth (Oxoid, UK) and cultured at 37°C overnight for genomic DNA extraction using the QIAamp DNA minikit (Qiagen, USA), and libraries (~500 bp inserts) were prepared with the Nextera DNA Flex Library Prep Kit (Illumina Inc., USA), following the manufacturer’s instructions. Sequencing was performed on an Illumina MiSeq platform (Illumina Inc., USA) (300 bp pair-end), yielding 3,328,020 reads. FastQC (v0.12.1) was used to assess data quality (5), followed by adapter removal, quality filtering, and trimming using FastP (v0.24.3) (6). Assembly was performed with Unicycler (v0.5.1) (7), and completeness was assessed with BUSCO (v5.8.0) (8). Phylogenetic identification was conducted with Type Strain Genome Server (v401) (9), and genome annotation with NCBI Prokaryotic Genome Annotation Pipeline (v6.10) (10). Antibiotic resistance genes, mobile genetic element (MGE), and prophages were detected using ResFinder (v4.7.2) (11), VRprofile2 (v2.0) (12), and PHASTEST (v3.0) (13), respectively. Additionally, BacAnt (v3.3.3) was employed to identify resistance genes, transposons, and insertion elements (14). Default parameters were applied, unless otherwise mentioned.

The final assembled draft genome sequence of *A. pittii* Vector-K19 was 3,769,256 bp in length and composed of 49 contigs, with an *L*_50_ value of 6, an *N*_50_ value of 235,347, a GC content of 39%, and a genome completeness of 99.6%, containing 3,521 coding sequences, 3,512 genes (with protein), and 60 tRNAs. Phylogenetic analysis based on genome BLAST distance phylogeny approach revealed that the isolate was most closely similar to *A. pittii* CIP 70.29. The genome’s resistome consisted of 11 antibiotic-resistance genes, mainly on two MGEs, ISAba43 and ISAba2/IS17. The presence of ISAba43 in *A. pittii* was noteworthy, as it has mostly been found in *A. baumannii*. One transposon, Tn6205, typically found in *A. baumannii*, was also identified. Prophage screening revealed two complete phage-related sequences, including PHAGE_Psychr_pOW20_A_NC_020841, which has not been previously reported in *A. pittii*.

## Data Availability

The raw reads utilized for the draft genome sequence assembly have been deposited in the Sequence Read Archive (SRA) (SRA accession number: SRR33784450) under BioProject number PRJNA1270812. The whole-genome shotgun project is available at DDBJ/ENA/GenBank with accession number JBOZRF000000000.
